# Possible Risk Reduction in Esophageal Cancer Associated with MPO -463 A allele

**DOI:** 10.2188/jea.11.109

**Published:** 2007-11-30

**Authors:** Keitaro Matsuo, Nobuyuki Hamajima, Masayuki Shinoda, Shunzo Hatooka, Manami Inoue, Toshiro Takezaki, Hiroyuki Onda, Kazuo Tajima

**Affiliations:** 1 Division of Epidemiology and Prevention, Aichi Cancer Center Research Institute.; 2 Department of Rehabilitation Service, Aichi Cancer Center Hospital.; 3 Department of Thoracic Surgery , Aichi Cancer Center Hospital.; 4 Department of Hospital Laboratory, Aichi Cancer Center Hospital.; 5 Department of Epidemiology, Nagoya University Graduate School of Medicine.

**Keywords:** esophageal cancer, genetic predisposition of disease, MPO, myeloperoxidase, polymorphism

## Abstract

Myeloperoxidase (MPO), an enzyme found in lysosomes of phagocytes, causes hydroxy radicals linked to DNA damage and activation of smoking related carcinogens. A -463 G/A polymorphism in the promoter region of the MPO gene results in reduced gene expression, which would imply lower susceptibility of esophageal cancer in mutant carriers. We conducted case-control study to test this hypothesis. Cases were 91 patients with esophageal cancer and controls were 241 non-cancer outpatients. MPO genotypes were examined by PCR-RFLP. The allele frequency for MPO -463A was found to be 8.2% for cases and 10.5% for controls. The age, sex, smoking and drinking status adjusted odds ratio for all subjects for MPO -463 GG/GA as compared to the AA was 0.61 (95% Cl: 0.28-1.32). The adjusted odds ratio for the GG/GA genotype was significantly low (0.15; 0.03-0.76, P=0.022) for those aged 61 years or older who had a significantly higher odds ratio for smoking than younger subjects. No difference was observed in disease risk when prevalent and incident cases were compared. Although there are limitations for interpretation of this study because of prevalent case-control study and partial statistical significance, these results suggest that MPO -463 A allele reduce the risk of esophageal cancer.

## INTRODUCTION

A number of epidemiological studies have revealed a close relationship between smoking and esophageal cancer^1^^)^ and investigations of the molecular epidemiology have been conducted to reveal the relations between genetic predispositions and environmental exposure. Most target gene polymorphisms with influence were found to be smoking related in enzymes such as cytochrome P450s (CYP1A1 and CYP2E1)^2^^-^^7^^)^ and glutathione S-transferases (GSTM1, GSTT1, and GSTP1)^5^^, ^^6^^)^.

Myeloperoxidase (MPO), an enzyme found primarily in lysosomes of phagocytes^8^^)^, catalyzes the reaction of chloride and hydrogen peroxide to yield hypochlorous acid that generates hydroxy radicals, a highly reactive radical species, in the presence of superoxide^9^^)^. MPO and its reactive by-products have been linked to generation or activation of carcinogens such as benzo(a)pyrene and aromatic amines^10^^, ^^11^^)^, DNA strand breakage^12^^)^ and inhibition of DNA repair^13^^)^. The biological evidence thus suggests a probable association with carcinogenesis.

A -463 G/A polymorphism, first described by Austin et al. in the promoter region of the MPO gene in acute leukemia cells^14^^)^, is commonly observed among the general population^15^^-^^17^^)^. The mutant type exhibits reduced mRNA expression^14^^, ^^18^^)^, resulting in decreased enzyme activity. Based on this observation and the contribution of MPO to metabolic activation of benzo(a)pyrene and aromatic organic compounds, the polymorphism could be important with reference to malignant diseases that are etiologically associated with smoking. Previously, the associations with aerodigestive tract cancers have been investigated^15^^-^^17^^, ^^19^^)^ and protective effects in mutant allele carriers regarding lung and laryngeal cancers. We hypothesized that the same might also be the case for esophageal cancers.

In the present study, we therefore conducted a hospital-based case control study to evaluate any association between esophageal cancer and the MPO polymorphism.

## MATERIAL AND METHODS

### Subjects

A total of 91 Japanese esophageal cancer patients (76 males and 15 females; median age, 61; age range, 43-76) and 241 non-cancer controls (118 males and 123 females; median age, 58; age range, 39-69) who visited Aichi Cancer Center in 1999 were recruited. Cases were firstly diagnosed as having esophageal cancers between 1984-2000. All subjects who gave written informed consent to participate in this study completed the self-administered questionnaire and provided blood. The questionnaire included the smoking status and alcohol consumption. Smoking status was classified into smoker, ex-smoker, and never-smoker, and the level of exposure was expressed in pack-years. Alcohol consumption was classified into three categories, never or occasional drinker, 1-4 times/week drinker and more than five times/week drinker.

### Genotyping procedure

DNA was extracted from the buffy coat fraction using a QIAamp blood mini kit (Qiagen Inc., Valencia, CA). MPO mutations were characterized by PCR-RFLP (polymerase chain reaction-restriction fragment length polymorphism) method as previously described^15^^-^^16^^)^. A 350-bp DNA fragment was amplified with MPOF (5′-CGG TAT AGG CAC ACA ATG GTG AG) and MPOR (5′-GCA ATG GTT CAA GCG ATT CTT C) primers. The setting for the thermal cycler were 35 cycles of 30s at 94°C, 30s at 56°C and 30s at 72°C, followed by 7min of extension at 72°C. PCR products were digested with Acil (New England Biolab, Schwalbach, Germany). The -463 G allele produced 289- and 61-bp, and the -463 A allele 168-, 121-, and 61-bp fragments which were identified by ethidium bromide staining of 3% agarose gels after electrophoresis.

### Statistics

All statistical analyses were performed with the STATA version 6 program (STATA Corporation, College Station, TX, USA). The expected allele frequency was calculated using the chi-square test for the Hardy-Weinberg law of equilibrium and odds ratios (ORs) were estimated using the unconditional logistic regression model. The factors employed for multivariate analyses were as follows: age; sex; smoking status, never smoker, ex-smoker, smoker (less than 50 pack-years), smoker (more than 50 pack-years); alcohol consumption, less than 5 times /week and more than 5 times.

This study was approved by the Institutional Review Board of Aichi Cancer Center.

## RESULTS

Table 1 summarizes subject data. The age distribution was slightly older among cases. Heavy smokers (>50 pack-year) constituted 33.0% of cases and 6.2% of the controls. Alcohol was also more frequently consumed by cases than by controls.

**Table 1.** Characteristics of cases and controls.

Characteristic		Cases (%) N=91	Controls (%) N=241
Age at diagnosis/interview	≤ 50	13 (14.3)	55 (22.8)
	51- 60	31 (34.0)	92 (38.2)
	61- 70	37 (40.7)	94 (39.0)
	71 -	10 (11.0)	0 ( 0.0)
Year from diagnosis	< 3	58 (63.7)	-
	≥ 3	33 (36.3)	-
Smoking status			
	Never	12 (13.2)	140 (58.1)
	Ever	21 (23.1)	46 (19.1)
	Smoker	58 (63.7)	55 (22.8)
	P-Y^a)^ ≤ 50	28 (30.7)	40 (16.6)
	P-Y> 50	30 (33.0)	15 ( 6.2)
Alcohol drinking			
	≥ 5 day /week	69 (75.8)	59 (24.5)
	< 5 day/week	22 (24.2)	182 (75.5)

^a)^ P-Y indicates pack-years.

The mutant allele frequencies for controls and cases were 10.6% and 8.2%, and the genotype distribution among controls was in accordance with the Hardy-Weinberg law of equilibrium; GG, 79.7%; GA, 19.5%; AA, 0.8%. The distribution among cases was GG, 83.5%; GA, 16.5%; AA, 0.0%.

As shown in Table 2, the crude odds ratio for the mutant genotype (GA and AA) relative to homozygous wild type (GG) was 0.81 (95% confidence interval (CI): 0.43-1.53, p=0.51). The age, gender, smoking status, and alcohol consumption adjusted odds ratio was 0.61 (0.28-1.32, P=0.21). Crude odds ratios by years from diagnosis were not different, 0.83 (0.39-1.76) for the cases whose year from diagnosis was less than three years and 0.71 (0.26-1.95) for the cases who were diagnosed three or more years before enrolment, therefore, we analyzed all cases together. Reduced odds ratios were observed for non-smokers, ex-smokers, and smokers with 50 pack-years or less, but not heavy smokers with more than 50 pack-years (see Table 3). Table 4 gives the odds ratios for lifestyle factors according to age group. The impact of smoking in subjects more than 61 years of age who showed reduced risk for MPO GA/GG type [adjusted odds ratio 0.15 (0.03-0.76)] was significantly high; odds ratios were 9.42 for ex-smokers, 13.8 for smokers with 50 pack-years or less, and 12.3 for smokers with more than 50 pack-years.

**Table 2.** Genotype frequencies of MPO and ORs.

	Genotype				
	N	GG(%)	GA(%)	AA(%)	GA/AA(%)
Cases	91	76 (83.5)	15 (16.5)	0 (0.0)	15 (16.5)
Controls	241	192 (79.7)	47 (19.5)	2 (0.8)	49 (20.3)
Crude OR		1.00	0.81	NE ^a)^	0.77
95%CI		Reference	0.43 -1.53	NE	0.41 -1.46
Adjusted OR^b)^		1.00	0.64	NE	0.61
95%CI		Reference	0.29 -1.41	NE	0.28 -1.32

^a)^ NE indicates not estimated.

^b)^ Odds ratios adjusted for age, sex, smoking status and alcohol consumption.

**Table 3.** Adjusted ORs and 95% CI for MPO GA/AA genotypes relative to MPO GG genotype.

Subjects	Genotype	Cases N=91	Controls N=241	Crude OR (95%CI)	Adjusted OR^a)^ (95%CI)
(Sex)					
Male	GG	64	93	1.00 (reference)	1.00 (reference)
	GA/AA	12	25	0.70 (0.33-1.49)	0.60 (0.25-1.47)
Female	GG	12	99	1.00 (reference)	1.00 (reference)
	GA/AA	3	24	1.03 (0.27-3.94)	0.74 (0.14-3.89)
(Age)					
≤ 60	GG	31	117	1.00 (reference)	1.00 (reference)
	GA/AA	13	30	1.64 (0.76-3.50)	0.95 (0.34-2.65)
≥ 61	GG	45	75	1.00 (reference)	1.00 (reference)
	GA/AA	2	19	0.18 (0.04-0.79)	0.15 (0.03-0.76)
(Smoking status)					
Non-smoker	GG	11	111	1.00 (reference)	1.00 (reference)
	GA/AA	1	29	0.35 (0.04-2.81)	0.32 (0.04-2.67)
Ex-smoker	GG	17	36	1.00 (reference)	1.00 (reference)
	GA/AA	4	10	0.85 (0.23-3.09)	0.51 (0.12-2.28)
Smoker					
P-Y ≤ 50	GG	26	32	1.00 (reference)	1.00 (reference)
	GA/AA	2	8	0.31 (0.06-1.58)	0.39 (0.07-2.23)
P-Y ≥ 50	GG	22	13	1.00 (reference)	1.00 (reference)
	GA/AA	8	2	2.36 (0.43-12.9)	1.40 (0.17-11.6)
(Alcohol drinking)					
Occasional∼< 5 days /week	GG	19	149	1.00 (reference)	1.00 (reference)
	GA/AA	3	33	0.71 (0.20-2.55)	0.87 (0.22-3.41)
≥ 5 days /week	GG	57	43	1.00 (reference)	1.00 (reference)
	GA/AA	12	16	0.57 (0.24-1.32)	0.52 (0.20-1.34)

^a)^ Odds ratios adjusted for age, sex, smoking status and alcohol consumption.

**Table 4.** Adjusted^a)^ ORs and 95% CI for life-style factors with reference to the age group (60 Years ≤ vs ≥ 61 Years).

	Age <60	Age >61
(Smoking status)		
Non-Smoker	1.00 (reference)	1.00 (reference)
Ex-smoker	1.32 (0.33-5.22)	9.42 (2.04-43.5)
Smoker		
P-Y≤ 50	1.94 (0.54-6.90)	13.8 (2.93-66.7)
P-Y> 50	17.7 (4.08-76.4)	12.3 (2.18-69.3)
(Alcohol drinking)		
Occasional ∼< 5 days /week	1.00 (reference)	1.00 (reference)
≥ 5 days /week	11.1 (3.76-32.6)	4.29 (1.42-13.0)

^a)^ Adjusted for age, sex and the MPO polymorphism.

## DISCUSSION

In this study, we found the MPO -463 G/A polymorphism to be linked to esophageal cancer susceptibility, especially among the older age population more significantly affected by smoking. Compared with the GA/AA genotype, the GG genotype thus showed an approximately six times higher risk in those aged more than 60. The gender-specific effect, which was observed in a several of studies^19^^, ^^25^^)^, was not observed in this study, although the number of female cases was limited.

In the present study, because of a low incidence of esophageal cancer, we analyzed all cases including prevalent cases together. To examine the difference in attribution of the polymorphism between prevalent and incident cases, we examined the odds ratios according to the years from diagnosis to enrollment (less than three years vs. three years or more), and no difference was observed between them, suggesting that the odds ratio reflected mainly the risk, not the prognosis.

MPO is an enzyme that is primarily localized in phagocytes where it generates hydroxy radicals with carcinogenic potential^20^^)^. Moreover, MPO activates established carcinogens like benzo(a)pyren and aromatic amines^21^^)^. The -463 G/A polymorphism is located in the promoter region of the MPO gene, where the Alu region with a hormone-responsive element binding the transcription factor SP1, highly relevant for promoter activity, is located^18^^)^. The G variant in this polymorphism shows a remarkable 25-fold higher transcription^18^^)^. This implies much lower enzyme activity and reduced cancer risk in A variant carriers.

Smoking related polymorphisms in genes encoding enzymes such as CYP1A1 or GSTM1 were previously examined with reference to esophageal cancer^3^^-^^7^^)^. Benzo(a)pyrene can be transformed to highly reactive intermediates by CYP1A1^22^^)^, but the studies failed to show any significant association between CYP1A1 and esophageal cancer among Caucasians and Japanese. GST(glutathione S-transferase), involved in detoxication, is known to have a relation to esophageal cancer^6^^-^^7^^)^. Although the MPO GA/AA type in the present study was associated with significant reduction of risk in the older group with higher odds ratios for smoking, no statistically significant effects were evident for the younger group. Carcinogenesis is considered as accumulation of genetic events during aging, and esophageal cancer has a steep slope for age-specific increase in incidence^23^^)^. The lower risk with the GA/AA genotype observed among elderly people in the present study implies that reduced activity of MPO contributes to a decreased incidence of genetic events in the aged, while the biological findings on the onset age and esophageal carcinogenesis are very scarce.

This study used 91 cases and 241 controls. The numbers of cases were similar to previous molecular epidemiological studies for esophageal cancer, and the numbers of controls was the larger than most of previous ones^2^^-^^7^^)^.

Several case-control studies have pointed to associations between the MPO polymorphism and lung^15^^-^^18^^)^ as well as laryngeal cancers^17^^)^, chronic granulomatous disease^24^^)^ and Alzheimer disease^25^^)^. In all cases, A variant carriers had reduced disease risk. To our knowledge, ours is the first study to document an association between esophageal cancer risk and the MPO polymorphism.

In summary, this study revealed that esophageal cancer risk for individuals with the MPO -463 A allele was reduced, especially in the aged, although a part of the effect might be related to the prognosis. Further studies to confirm the association in prospectively collected populations and elucidate biological relations with other metabolic pathways are now required.
